# The effect of body mass index on global brain volume in middle-aged adults: a cross sectional study

**DOI:** 10.1186/1471-2377-5-23

**Published:** 2005-12-02

**Authors:** Michael A Ward, Cynthia M Carlsson, Mehul A Trivedi, Mark A Sager, Sterling C Johnson

**Affiliations:** 1 Geriatric Research Education and Clinical Center, Wm. S. Middleton VA Hospital, Madison, WI, USA; 2 Geriatrics and Adult Development, University of Wisconsin Medical School, Madison, WI, USA

## Abstract

**Background:**

Obesity causes or exacerbates a host of medical conditions, including cardiovascular, pulmonary, and endocrine diseases. Recently obesity in elderly women was associated with greater risk of dementia, white matter ischemic changes, and greater brain atrophy. The purpose of this study was to determine whether body type affects global brain volume, a marker of atrophy, in middle-aged men and women.

**Methods:**

T1-weighted 3D volumetric magnetic resonance imaging was used to assess global brain volume for 114 individuals 40 to 66 years of age (average = 54.2 years; standard deviation = 6.6 years; 43 men and 71 women). Total cerebrospinal fluid and brain volumes were obtained with an automated tissue segmentation algorithm. A regression model was used to determine the effect of age, body mass index (BMI), and other cardiovascular risk factors on brain volume and cognition.

**Results:**

Age and BMI were each associated with decreased brain volume. BMI did not predict cognition in this sample; however elevated diastolic blood pressure was associated with poorer episodic learning performance.

**Conclusion:**

These findings suggest that middle-aged obese adults may already be experiencing differentially greater brain atrophy, and may potentially be at greater risk for future cognitive decline.

## Background

The prevalence of overweight and obese people in the United States and other developed nations has progressively increased over the last twenty years and is now at epidemic proportions[1]. It is estimated that greater than 60% of all Americans are overweight, and approximately one-half of that group are classified as obese[2]. Previous studies have found that obesity reduces life expectancy [3] by causing or exacerbating various medical conditions including coronary heart disease (CHD), type 2 diabetes mellitus, hypertension, obstructive sleep apnea, and stroke[4]. Neurocognitive health may also be related to obesity. A recent study determined that obesity was strongly associated with poorer cognitive function in individuals over 65 years of age [5]. In a population-based sample of women aged 70–89 years, greater body mass index (BMI) in middle and later life was associated with cerebral white matter ischemic change [6], a higher incidence of dementia, particularly Alzheimer's disease (AD) [7], and temporal lobe atrophy [8] in later life.

Brain atrophy involves the loss of tissue volume and is commonly seen with increasing age [9-11] and neurodegenerative disease[12]. Vascular factors intrinsic to overweight individuals, such as hypertension[13,14], hypercholesterolemia [13,15], endothelial dysfunction[16,17], and diabetes [18-20] have all been linked to greater risk for dementia or brain atrophy in the elderly. Furthermore, older adults with better cardiovascular fitness demonstrate significant improvements in cognitive function and a significant slowing of age-related atrophy of gray and white matter[21]. Together these findings suggest that older overweight individuals have a higher risk of accelerated brain atrophy and concomitant cognitive decline.

While the deleterious effects of obesity on the brain in the geriatric population are now apparent, it is not known whether this relationship occurs in younger persons or is unique to older populations. This is an important question because interventions to reduce the adverse effects of obesity may have a larger public health impact when implemented at younger ages. The purpose of the present study was to determine whether the effect of BMI on brain atrophy previously observed in elderly females [8] might also be observable in cognitively healthy adults between the ages of 40 and 66, and to determine the relationships between this effect and associated cardiovascular factors (hypertension and hypercholesterolemia).

## Methods

One hundred seventeen participants (44 male, 73 female) with a mean age of 54.2 years (SD = 6.5) were studied with magnetic resonance imaging (MRI) and cognitive testing as part of a cross-sectional study analyzing factors related to global brain volume and cognition. Sixty-five participants were recruited from an existing registry known as the Wisconsin Registry for Alzheimers' Prevention (WRAP)[22] consisting of cognitively normal middle-aged adults who had at least one parent with AD. These participants were recruited to enrich the sample with individuals having risk factors for AD. The remaining fifty-two participants were recruited somewhat simultaneously from the University of Wisconsin-Madison (UWM) community. This convenience sample was selected to have no known first-degree family history of AD (with parents surviving until at least age 70 without dementia). All participants in this study were required to be between the ages of 40 and 66 and have no current major Axis I psychiatric disease or history of major medical conditions (i.e., traumatic brain injury, neurovascular infarctions, brain neoplasms or ischemic changes, history of cancer, diabetes, or condition requiring an invasive brain procedure). Additionally, participants were required to have normal cognitive function, and MRI scanner compatibility. Lastly, participants on any medication with potential to affect cerebral perfusion or cognition (such as beta blockers, calcium channel antagonists, Angiotensin-converting Enzyme (ACE) inhibitors, statins, or Selective Serotonin Reuptake Inhibitors (SSRIs)) were excluded from the analysis.

All participants completed a detailed health history questionnaire, and were administered a battery of neuropsychological tests and laboratory blood tests. Data were collected on Apolipoprotein E (APOE) genotype, non-fasting total blood cholesterol level, blood pressure (BP), height and weight (for BMI calculation). BP was measured with the subject seated and at rest using an automated BP machine. Body height and weight were collected to the nearest 0.5-inch and one pound respectively. The battery of neuropsychological tests [23] included the following: portions of the Wechsler Adult Intelligence Scale-Third Edition (WAIS-III), the Rey Auditory Verbal Learning Test (RAVLT), Trail Making Test A and B, and the Center for Epidemiological Studies Depression (CES-D) Inventory. All participants gave written informed consent under a protocol approved by the local institutional review board. This study was performed in a manner that was in accordance with the Declaration of Helsinki.

### Brain imaging

MRI was performed using a General Electric 3.0 Tesla SIGNA (Waukesha, WI) MRI system. A 3D IR-prepped fast gradient echo pulse sequence provided high-resolution T1-weighted structural images. The whole brain was imaged in the axial plane with the following parameters: inversion time = 600 ms, fast gradient echo read-out with TR/TE/flip = 9 ms/1.8 ms/20°; acquisition matrix = 256 × 192 × 124 (interpolated to 256 × 256 × 124); field of view = 240 mm; slice thickness = 1.2 mm (124 slices); ± 16 kHz receiver bandwidth.

A Fast Recovery Fast Spin Echo 2D T2-weighted axial sequence was also acquired with the same start and stop locations as the T1 weighted images. The parameters were: field of view = 240 mm, matrix 256 × 256 × 64, TR = 9000 ms, TE = 93 ms, flip angle = 90. Seventy slices were acquired; slice thickness = 1.7 mm with 0.3 mm skip. An experienced neuroradiologist examined all images for evidence of any neurovascular disease or structural abnormality that would exclude the subject from the analysis (see exclusions above).

### Global brain atrophy determination

Global brain volumes, calculated from T1-weighted images, were analyzed using the cross sectional method of Structural Image Evaluation, using Normalization, of Atrophy (SIENAX) within the FSL 3.1β software suite[24]. The SIENAX parameters were set for a three-class segmentation of tissue type and at a 0.3 threshold for segmenting the brain from extra-axial soft tissue. SIENAX analysis yielded whole-brain volumetric data, in units of cubic millimeters, for three different tissue types: cerebral spinal fluid (CSF), gray matter, and white matter. Total intracranial volume (TICV) was defined as the sum of the three tissue types, and whole brain parenchyma volume (BV) was defined as the sum of gray matter and white matter. BV was divided by TICV to obtain a normalized brain volume (NBV) with respect to head size.

### BMI classification

BMI values were calculated in standard fashion by dividing the weight in kilograms by the square of height in meters. BMI groups were defined using the World Health Organization's (WHO) classification system: Underweight, less than 18.5; Normal, 18.5–24.9; Overweight, 25.0–29.9; and Obese, greater than 30.0 (in units kg/m^2^) [25]. There were not enough underweight participants in this study group to analyze the effects of underweight on global brain atrophy. Therefore, individuals classified as underweight were not included in the statistical analysis.

### Statistical analysis

First, Student's t-tests were performed to determine whether the participants differed by family history of AD status/referral source. These analyses revealed that UWM participants were more highly educated than the WRAP participants, mean = 16.9 (SD = 2.4) and mean = 15.9 (SD = 2.5) respectively (t [112] = -2.10; p= 0.038). In addition, the frequency of the APOE ε4 allele was more prevalent in WRAP participants (54.0 %) compared to the UWM participants (13.7 %) (chi-square [1] = 23.5; p < 0.001). No other variables were found to be significantly different between the two groups. Nevertheless, 1^st ^degree family history of AD was treated as an independent variable in the ensuing stepwise regression model.

Next, linear regression analyses and stepwise regression analyses were used to predict the effect of BMI, age, non-fasting total blood cholesterol level, systolic and diastolic BP, family history of AD, gender, education, and genotype on the dependent variables of NBV and cognition in separate analyses. Family history of AD, gender, and APOE genotype were entered into the model as dichotomous variables: history/no history of AD, male/female, and presence/absence of the ε4 allele, respectively. A method advocated by Baron et al. [26] was used to determine whether any variable within the stepwise linear regression model mediated the effect of BMI on NBV.

Three participants were not included in the brain volume analysis. Two participants were classified as underweight and one participant was discovered to have a previously undiagnosed brain tumor. Therefore the final sample size equaled 114 participants. Nine participants were not included in the cognition analysis; five participants scored a fifteen or higher on the CES-D inventory suggesting symptoms of depression, one participant did not complete the CES-D inventory, and the remaining three participants were the same participants excluded in the brain atrophy analysis (n= 108 participants). Therefore the final sample size for the cognition analysis equaled 108 participants.

## Results

One hundred fourteen middle-aged (mean age 54) adults were administered a comprehensive battery of neuropsychological tests and structural MRI scans in this cross-sectional study examining the brain and cognitive effects associated with various AD risk factors. Baseline demographics, cognitive measures, and predictor variables are shown in Tables 1 and 2.

**Table 1:** Demographic and cognitive measures for men and women

Demographic and Cognitive Variables	Mean (SD)
Age (years)	54.2 (6.6)
Education (years)	16.4 (2.5)
Classification: n = Normal / Overweight / Obese^a^	51 / 42 / 21
Trail Making Test B (seconds)^b^	61.3 (20.2)
WAIS-III-Digit Span raw score^b^	17.9 (3.8)
RAVLT-Total raw score^b^	49.6 (7.9)
CES-D^b^	4.6 (5.2)

Data are presented as mean (standard deviation).

Notes: ^a^Normal, overweight, and obese body types are defined using the World Health Organization's classification system.

^b^The cognitive and mood variables are presented for the 108 participants in the cognitive analysis. Demographic variables are for the full sample of 114.

**Table 2:** Independent associations between each predictor variable and age-adjusted NBV

Regressor	Mean (SD)	Range	β-value	p-value
BMI (kg/m^2^)	26.0 (4.4)	(19.0–39.7)	-0.22	0.010
Total Cholesterol (mg/dL)	208.0 (37.8)	(114–339)	-0.12	0.178
Systolic BP (mm Hg)	132.4 (17.2)	(102–205)	-0.15	0.092
Diastolic BP (mm Hg)	79.4 (10.4)	(54–110)	-0.09	0.328
Family History AD (y/n)	63/51		0.07	0.448
APOE Genotype (ε4/no ε4)	41/73		-0.04	0.660
Gender (Male/Female)	43/71		0.13	0.137

n = 114. β-values, acquired using individual linear regression analyses with each of the above predictors entered after age, reflect age-adjusted predictions of NBV.

**Figure 1:**
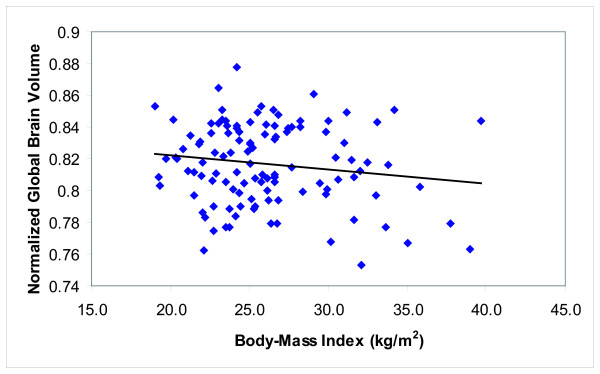
**NBV versus BMI**. Age adjusted values of NBV plotted versus BMI scores. NBV is shown to decrease proportionally with increasing BMI (*r *= -0.232, p < 0.03).

An initial linear regression analysis examined the effect of age and BMI on NBV to determine whether the effect observed in elderly females [8] could be observed in younger men and women. This analysis indicated that both age (β = -0.390; t = -4.614; p < 0.0001) and BMI (β = -0.220; t = -2.605; p = 0.010) were significant predictors of NBV (*R*^2 ^= 0.206; F [111, 2] = 14.432; p < 0.00001). Figure 1 shows that elevated BMI is associated with decreased NBV (after adjusting for age). To determine whether additional variables may also predict NBV, a stepwise linear regression analysis was used to examine the effects of age, BMI, family history of AD, APOE genotype, total cholesterol, systolic and diastolic BP, and gender on NBV. This analysis found that age (β = -0.389; t = -4.337; p < 0.001) and BMI (β = -0.224; t = -2.495; p < 0.014) together were the best predictors of NBV (*R*^2 ^= 0.205; F [99, 2] = 12.756; p < 0.0001). No other variables were significant.

Pearson correlations determined that BMI was significantly associated with cholesterol (*r *= 0.21, p < 0.04) and systolic BP (*r *= 0.36, p < 0.001) but was not associated with any other predictor variable. Neither cholesterol nor systolic BP were significantly associated with NBV (results are summarized in Table 2), and therefore did not appear to mediate the effect of BMI on NBV according to criteria specified by Baron et al. [26].

Next we applied the same stepwise model above, including education to predict the non-crystallized cognitive abilities of learning, processing speed and working memory in separate regression models. Age (β = -0.315; t = -3.417; p < 0.001) and diastolic BP (β = -0.233; t = -2.526; p = 0.013) were significant predictors of learning (*R*^2 ^= 0.157; F [101, 2] = 9.252; p < 0.001). Age was the only significant predictor of processing speed (*R*^2 ^= 0.081; F [100, 1] = 8.454; p < 0.01). There was no significant predictor of working memory in this sample. Average neuropsychological scores are given in Table 1.

## Discussion

This study found that elevated BMI is associated with reduced brain volumes, suggesting greater global brain atrophy in middle-aged adults even after adjusting for age. Episodic learning, working memory, and processing speed abilities were not associated with BMI in this sample indicating that the possible effect on cerebral atrophy had not influenced cognition in participants with a high BMI. Episodic learning was related to diastolic BP, which indicates that certain cardiovascular risk factors may be associated with cognition. However, this finding was isolated to only one of our three categories of cognition. Longitudinal studies are needed to assess future cognitive consequences associated with cardiovascular risk factors.

Our finding that BMI affects brain volume at a relatively young age extends prior research on the harmful effects of obesity on the brain. We found a 2.4% difference in brain parenchyma volume for participants classified by the WHO as obese (n = 21 people; average BMI= 33.3 kg/m^2^) compared to participants at a normal, healthy weight (n = 51 people; average BMI = 22.6 kg/m^2^). Although the magnitude of this difference is not large in this cross-sectional sample, when we consider these results in the context of the younger age of our sample (54.2 years), it follows that obesity in middle age may render an individual more vulnerable to brain atrophy over subsequent years, and thus more vulnerable to cognitive decline or dementia. The results of this study are consistent with others showing that life choices (i.e., proper diet, physical activity) may be neuroprotective and, therefore, potentially reduce the rate of brain atrophy and concomitant cognitive decline [27,28]. A longitudinal study of obese people at middle age is needed to test these hypotheses.

It is unclear through what mechanisms obesity affects brain volume. Central obesity is associated with risk factors composing the metabolic syndrome, including high triglyceride levels, low HDL cholesterol levels, hypertension, insulin resistance, and prothrombotic and proinflammatory states. Prior studies indicate that some of these risk factors such as mid-life elevations in blood pressure, total cholesterol, and inflammatory markers, are risk factors for late-life dementia[13,29-31]. In the present study, neither BP nor non-fasting total cholesterol was associated with current NBV (though systolic BP did show a trend). These vascular risk factors may affect chronic cerebral perfusion and β-amyloid generation, thus influencing neuronal degeneration. Leptin, a peptide related to obesity, also affects β-amyloid regulation [32]. Further studies are needed to determine the mechanism by which BMI affects brain atrophy in middle-aged adults, and whether this may have future deleterious consequences on brain structure or function.

Brain tissue is strongly dependent upon oxygen for survival and is thus vulnerable to hypoxic and ischemic conditions. Hypoxia is an inherent consequence of many of the conditions caused or exacerbated by obesity. Obstructive sleep apnea[4], CHD [33], asthma [34], and low cardiovascular fitness[21] may lead to hypoxia which, in chronic situations, may lead to cognitive decline and neuronal death[35]. Hypoxia is often a consequence of hypoperfusion, which is strongly associated with endothelial dysfunction common to individuals with hypertension, atherosclerosis, and CHD. Endothelial dysfunction, as a result of oxidative stress, may induce neuronal damage and initiate neurodegenerative change[17].

Prevention or postponement of the onset of dementia has the potential to drastically impact the prevalence of dementia. The prevalence of AD in the United States alone is projected to quadruple in the next 50 years, and if disease onset could be delayed by only one year, it would result in 800,000 fewer cases[36]. It has been shown that a person's weight is a reflection of their habitual physical activity[4]. Given the rapid increase in obesity in the United States, targeting weight management may significantly impact the prevalence of dementia. High dietary fat intake has been shown to increase the risk of dementia [37]. Physical fitness significantly improves cognitive function in older adults[21] and exercise increases the production of brain-derived neurotrophic factor (BDNF) and insulin-like growth factor I (IGF-I). BDNF and IGF-I are neurotrophic hormones that are important for neurogenesis, which may protect against neurodegeneration seen with aging[38]. In older adults the rate of change in brain volume may be diminished in persons with better cardiovascular fitness[39].

There were some limitations of this study. First, the range of BMI was truncated because of the small-bore radius (55 cm) inherent to the MRI scanner. Therefore, participants in this study could not exceed 260 pounds, which did not permit an analysis of the effects of extreme obesity. Next, insulin-dependent diabetic participants were excluded from this study because diabetes has been shown to be a risk factor for dementia [18-20] and therefore could be an important confounder in the analysis. The incidence of diabetes in our study population was less than 1%, which was not enough to determine its affect on global brain volume. Next, the design of this study was cross-sectional which only allowed for inferences between participants over a single time point. A longitudinal study is needed to clarify if the reduced brain volume associated with elevated BMI is representative of a progressive brain atrophy and concomitant cognitive decline. Additionally, non-fasting, instead of the more accurate fasting blood cholesterol values were used as potential predictors of NBV. Furthermore, the BMI measurement itself is only an estimate of obesity; it does not accurately distinguish fat mass from lean mass. The highest risk for adverse consequences related to overweight individuals are best indicated by excess amounts of adipose tissue, especially that within the intra-abdominal region. Waist circumference and skinfold thickness measurements in conjunction with BMI would have provided a more precise measure of the amount and location of this excess body fat. Finally, this study population was self-selected from a group of highly educated, motivated volunteers; these findings may not be representative of the general population. However, these findings are important in understanding how to potentially target preventive therapies for at-risk individuals.

## Conclusion

This study found that elevated BMI is associated with lesser brain volume in middle-aged adults (mean age = 54 years) even after adjusting for age. This study extends prior research that indicated a similar effect in a population-based sample of females aged 70 to 84 years with elevated BMIs from middle age through later life (46 to 84 years). Further, longitudinal studies are needed to determine whether the effect we have observed at middle age results in greater rate of cerebral atrophy over time, and whether this effect may increase the risk of future cognitive decline and incidence of dementia.

## Competing interests

The author(s) declare that they have no competing interests.

## Authors' contributions

MAW carried out the statistical analysis, drafted the manuscript, and assisted in the acquisition of data. CMC contributed to study conception, participated in its design, and assisted in drafting the manuscript. MAT assisted in data acquisition, statistical analyses, interpreting the results, and drafting the manuscript. MAS assisted in data acquisition, interpreting the results, and writing the manuscript. SCJ drafted the manuscript, conceived of the study concept and design, assisted in the statistical analysis, and interpretation of the results. All authors read and approved the final manuscript.

## Pre-publication history

The pre-publication history for this paper can be accessed here:


